# Effect of Hydrothermal Dewatering on Low-Temperature Oxidation of Lignite

**DOI:** 10.3390/molecules30091932

**Published:** 2025-04-26

**Authors:** Qiong Mo, Junjie Liao, Yankun Yang, Lin Gao, Liping Chang, Weiren Bao, Xianshu Dong, Yuping Fan, Guichuan Ye

**Affiliations:** 1 College of Mining Engineering, Taiyuan University of Technology, Taiyuan 030024, China; dongxianshu@tyut.edu.cn (X.D.); fanyuping@tyut.edu.cn (Y.F.); guichuanye@hotmail.com (G.Y.); 2 Shanxi Research Institute for Clean Energy, Tsinghua University, Taiyuan 030032, China; 3 State Key Laboratory of Clean and Efficient Coal Utilization, College of Chemistry and Chemical Engineering, Taiyuan University of Technology, Taiyuan 030024, China; 18406556513@163.com (Y.Y.); gl1138175@163.com (L.G.); lpchang@tyut.edu.cn (L.C.); baoweiren@tyut.edu.cn (W.B.); 4 Key Laboratory of Coal Science and Technology, Ministry of Education, Taiyuan University of Technology, Taiyuan 030024, China

**Keywords:** lignite, hydrothermal dewatering, low-temperature oxidation, kinetic analysis, correlation analysis

## Abstract

The hydrothermal dewatering (HTD) of lignite results in noticeable variations in the low-temperature oxidation process. Consequently, this study was made on the gas release and temperature change characteristics to investigate the oxidation kinetics and mechanism of HTD coal samples. In this study, a lignite from Inner Mongolia in China was upgraded by HTD. N_2_ adsorption, SEM, FT-IR, and chemical titration experiments were also carried out on raw and HTD coal samples to relate the physico-chemical structure properties with low-temperature oxidation characteristics. Results show that HTD coal samples have higher low-temperature oxidation activities and lower critical ignition temperatures compared with raw coal. According to the change in activation energy by kinetic analysis, the low-temperature oxidation process in the temperature range 35–140 °C could be divided into the stage I (oxygen adsorption stage) and stage II (accelerated oxidation stage). The correlation analysis indicates that the oxygen adsorption stage is controlled by the aliphatic and surface structures, while the accelerated oxidation stage is jointly affected by the competition of physico-chemical structures. The oxygen adsorption stage promotes the progress in accelerated oxidation stage.

## 1. Introduction

Coal serves as the primary energy resource in China. With the large consumption of high-rank coal, low-rank coal, especially lignite, has attracted wide attention because of its abundant reserves, low mining cost, high reactivity, high volatile content, and less impurities. However, lignite features high moisture content, which could cause excess transporting and operating costs. It is usually necessary to dewater and upgrade lignite before utilization [1,2]. Hydrothermal dewatering (HTD) is a non-evaporating technology, in which water in lignite is removed in a liquid state, preserving the latent heat of vaporization. Lignite is added into the autoclave and heated to 150–350 °C, along with additional water for providing saturated vapor pressure during this process. HTD can change the physico-chemical properties of lignite significantly, avoiding the upgraded yields from the reabsorption of water [3,4,5,6]. The lignite after HTD is still active, which makes it easy to for low-temperature oxidation, further resulting in spontaneous combustion [7]. It brings potential security risks for storage and transportation. Therefore, this study mainly focuses on the low-temperature oxidation behavior of HTD lignite in order to provide theoretical guidance for large-scale lignite utilization.

Thus far, the oxidation of lignite at low temperatures has been extensively investigated. The low-temperature oxidation of lignite is a complicated process, accompanied by a series of variations, such as mass, heat, and release of gaseous products [8,9,10,11]. Among them, the release of gaseous products is a typical macroscopic characteristic of the oxidation process of lignite. When lignite makes contact with O_2_, oxidation reactions occur in the active groups of lignite and produce unstable intermediates. Then, a mass of gaseous products are released following the oxidation and thermal decomposition reactions. The gaseous products mainly include CO, CO_2_, CH_4_, and C_2_H_6_ [12,13,14,15]. From the point of view of the amount of gas released, the main gases released from lignite are CO and CO_2_, which mainly come from the decomposition of not only surface oxides, caused by the reaction between O_2_ and active groups in coal, but also the inherent oxygen-containing functional groups in the coal matrix [15,16,17]. The aliphatic structures such as –CH_3_ and –CH_2_– are key active groups that affect the initial process of low-temperature oxidation [18]. Based on the variation law of gaseous products, the low-temperature oxidation process of coal can be divided into different stages. The gas release behaviors are different in different stages, because the gases are generated from different reactions. Therefore, the low-temperature oxidation process is a phased process [19]. The reaction mechanism of low-temperature oxidation and the influencing factors for the reaction rate are explored by establishing a kinetic model and calculating kinetic parameters, such as the apparent activation energy *E*_α_ [19,20,21]. The low-temperature oxidation process can be segmented according to the change in activation energy, and the influencing factors of each stage can be correspondingly studied. The low-temperature oxidation process is divided into the oxygen adsorption stage (30–140 °C) and the oxygen desorption stage (140–300 °C) according to the TG curve of the low-temperature oxidation process (30–300 °C) [10]. The low-temperature oxidation process is also divided into the slow oxidation stage (30–95 °C), accelerated oxidation stage (about 95–150 °C), and rapid oxidation stage (about 150–200 °C) [15]. The same conclusion is obtained by differentiating the DSC curves [11]. The activation energy in the reaction process is calculated based on the release rates of CO and CO_2_ in the process of constant temperature oxidation, and the calculation concludes that the two reactions come from two independent reaction channels [22]. As for the influencing factors for each stage, the slow oxidation stage is not affected by diffusion [23,24,25].

In terms of the low-temperature oxidation of dewatered lignite, Liao et al. tested the critical ignition temperatures of HTD coal samples with the basket heat method and found that the spontaneous combustion tendency of lignite increases after HTD [7]. The crossing point temperature and isothermal oxidation gas analysis methods are used to test the low-temperature oxidation activity of upgraded lignite, and it is found that the low-temperature oxidation reactivity and spontaneous combustion tendency decrease because of the coating of the asphalt on the coal surface [26]. The coal sample dewatered with hot gas has high low-temperature oxidation activity [27]. It can be seen that different upgrading methods and conditions have different effects on the low-temperature oxidation activity.

Generally speaking, the studies on low-temperature oxidation in the existing literatures mainly focus on the overall process [28,29,30,31]. The oxidation behavior in the low temperature range is usually ignored, which is the key factor for controlling spontaneous combustion. The inherent oxygen-containing functional groups of lignite will decompose when the test temperature increases to a certain level, which is not conducive to the exploration in the oxidation process. In addition, there is a possibility of spontaneous combustion for lignite at higher temperatures, which brings great security risks to the experiment. Therefore, the low-temperature oxidation process of lignite in the low temperature range of 35–140 °C is emphasized in this study. For the index, the CO_2_ is selected to evaluate the low-temperature oxidation activity of lignite because the CO_2_ is much easier to be detected at 35–140 °C. In order to analyze the low-temperature oxidation process of HTD coal samples and explore the mechanism of HTD on the low-temperature oxidation of lignite, the lignite from Inner Mongolia in China is upgraded by HTD at 170, 230, and 300 °C. The reason for choosing the above temperatures to prepare coal samples is that the functional groups, pore volume, and specific surface area of lignite change significantly during the HTD process [32].

**Figure 1:**
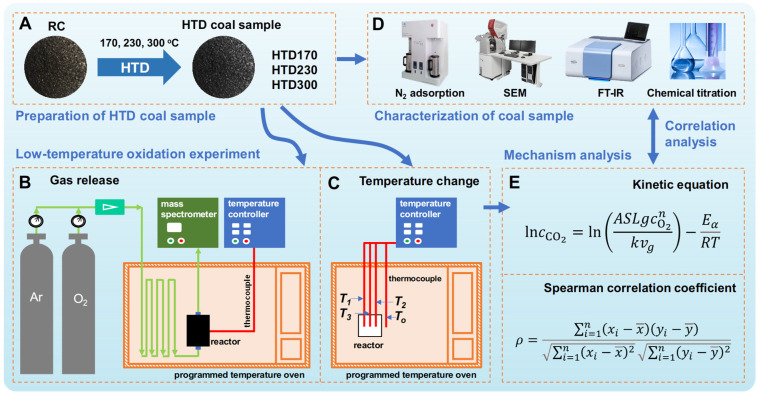
Schematic diagram of experimental procedure: preparation of HTD coal sample (**A**); low-temperature oxidation experiment for testing gas releasing (**B**); low-temperature oxidation experiment for testing temperature changing (**C**); characterization methods of coal samples (**D**); kinetic and correlation analysis equations (**E**).

This study aims to investigate the effect of HTD on the low-temperature oxidation of lignite. As shown in Figure 1, the gas release amount and temperature change in the HTD coal samples in the process of low-temperature oxidation were measured, and the kinetics of gas releasing and correlation analysis were investigated to explore the influencing factors on low-temperature oxidation and relate the structure of lignite with its low-temperature oxidation activity for prediction of the spontaneous combustion behavior of lignite by the regulation of coal structure.

## 2. Results and Discussion

### 2.1. Low-Temperature Oxidation Characteristics of HTD Coal Samples

**Figure 2:**
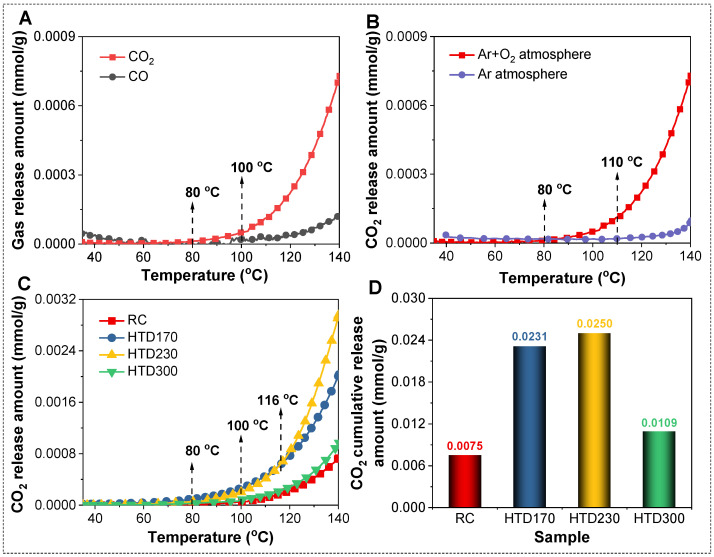
CO and CO_2_ release curves for RC (**A**). CO_2_ release curves for RC in different atmospheres (**B**). CO_2_ release curves for RC and HTD coal samples in low-temperature oxidation process (**C**). The cumulative release amounts of CO_2_ at 35–140 °C (**D**).

The release curves of CO and CO_2_ from raw coal (RC) during low-temperature oxidation from 35 °C to 140 °C are shown in Figure 2A. The figure displays that the CO and CO_2_ release amounts from RC are weak at 35–100 °C and increase significantly in an exponential form with the increase in oxidation temperature after 100 °C. The amount of CO_2_ released is obviously higher than that of CO. Figure 2B shows the CO_2_ release curves for RC under the simulated air and pure Ar atmospheres during the programmed heating process. It is found that the release of CO_2_ in the simulated air is significantly higher than that in pure Ar, indicating that CO_2_ mainly comes from the oxidation of lignite and a small part from thermal decomposition at 35–140 °C. Therefore, it is reasonable to select CO_2_ as the indicator to study the low-temperature oxidation activity of lignite in the temperature range of 35–140 °C.

The CO_2_ release behaviors of HTD coal samples during low-temperature oxidation were studied. As shown in Figure 2C, the CO_2_ release amounts of RC and HTD coal samples increase significantly with the increase in oxidation temperature after 100 °C, and the generating rate is getting faster and faster with the rising oxidation temperature. At room temperature, O_2_ contacts with lignite, and physical adsorption takes place on the lignite surface. As the temperature increases, the physical adsorption changes to chemical adsorption, and active groups on the lignite surface interact with O_2_ to form intermediates. For instance, the aliphatic structure –CH_3_ and –CH_2_– (CH_X_) would react with O_2_ to form hydroperoxides (–C–O–OH) and peroxides (–C(O)–O–OH) on the coal surface [15]. The above process mainly occurs at relatively low temperatures. Therefore, the amount of CO_2_ released is too weak to be detected in the system from room temperature to 100 °C. As the oxidation temperature increases to 100 °C, a series of chemical reactions occur and oxidation reactions are accelerated, resulting in the production of CO_2_, such as the thermal decomposition of surface oxides and inherent oxygen-containing functional groups. Different coal samples have different releasing behaviors. Compared with RC, all HTD coal samples release higher amounts of CO_2_ at 100–140 °C. HTD300 releases slightly more CO_2_ than RC. HTD170 and HTD230 release higher levels of CO_2_, but their changes are complex. At 80–116 °C, the amount of CO_2_ released from HTD170 is higher than that from HTD230. When the oxidation temperature is higher than 116 °C, the CO_2_ releasing order for HTD230 and HTD170 is inversed.

The CO_2_ release curve for each coal sample in the temperature range 35–140 °C during the low-temperature oxidation process was integrated to quantitatively analyze the cumulative amount of released CO_2_. As listed in Figure 2D, the CO_2_ cumulative release amounts for HTD coal samples are higher than that for RC, and show a trend of first increasing and then decreasing with the increase in the HTD temperature. The CO_2_ cumulative release amount for HTD230 is the largest (0.0250 mmol/g), 3 times higher than that for RC (0.0075 mmol/g). Compared to HTD230, HTD170 and HTD300 have lower CO_2_ cumulative release amounts (0.0231 and 0.0109 mmol/g, respectively). The above results indicate that HTD changes the low-temperature oxidation activity of lignite, and the influence degree is diverse for different HTD temperatures.

**Figure 3:**
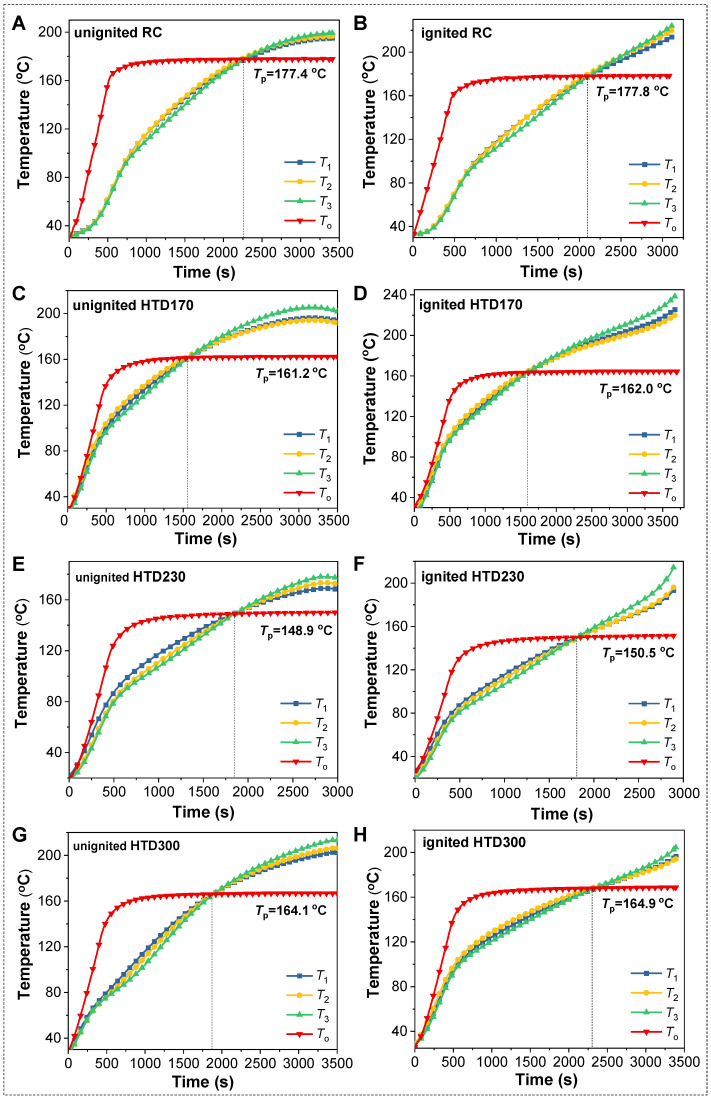
The temperature change in the RC and HTD coal samples in low-temperature oxidation and spontaneous combustion processes (**A**,**C**,**E**,**G**) for the unignited RC, HTD170, HTD230, and HTD300, respectively; (**B**,**D**,**F**,**H**) for the ignited RC, HTD170, HTD230, and HTD300, respectively.

In order to further figure out the low-temperature oxidation process, the temperature changes in RC and HTD coal samples were determined, as shown in Figure 3. Initially, the oven temperature (*T*_o_) increases rapidly until the set temperature is reached. The rapid increase in *T*_o_ raises the temperature of the coal. From Figure 3, we observed that the center temperature in coal sample (*T*_3_) is close to its left and right temperatures (*T*_1_ and *T*_2_). As the temperature increases, *T*_3_ gradually lags behind *T*_1_ and *T*_2_, because the heat transfer from the outside to the inside leads to a low center temperature. However, the gap between the center temperature and the surrounding temperature becomes smaller and smaller until they coincide with the oven temperature, and then the center temperature exceeds the surrounding temperature. This is due to oxidative heat release, where heat accumulates and increases the temperature of the center. When the coal temperature is the same as the oven temperature, it is called the cross-point temperature (*T*_p_). After the coal temperature exceeds the oven temperature, it will continue to rise due to the release of heat from oxidation.

**Figure 4:**
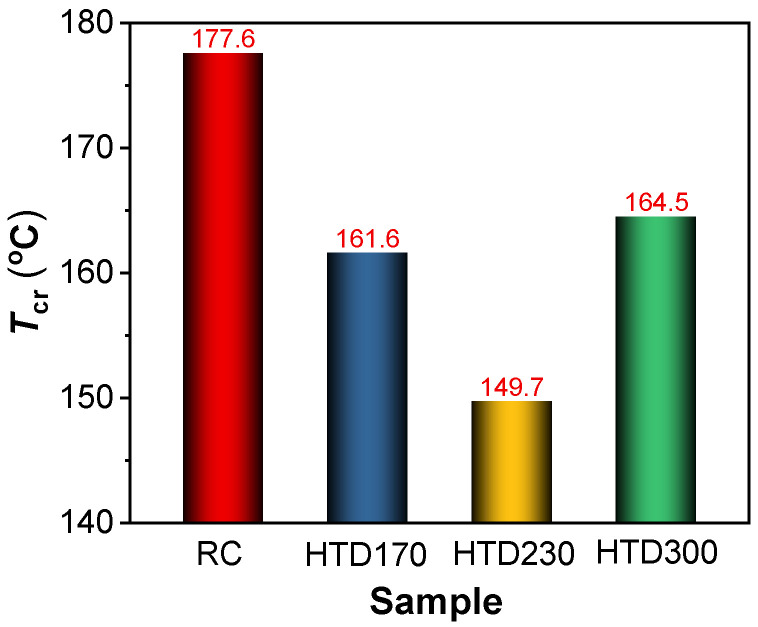
The critical ignition temperatures of coal samples.

In general, spontaneous combustion will not occur when the coal temperature cools down after reaching the highest temperature, as shown in Figure 3A,C,E,G. The cross-point temperatures are marked with *T*_p,un_. The continuous heat storage will lead to thermal runaway, and spontaneous combustion will occur, as shown in Figure 3B,D,F,H. The cross-point temperatures are marked with *T*_p,ig_. According to Formula (1), the critical ignition temperatures (*T*_cr_) of coal samples are calculated and listed in Figure 4.

(1) $$T_{cr}=\frac{{\left(T_{p,un}\right)}_{max}+{\left(T_{p,ig}\right)}_{min}}{2}$$

The *T*_cr_ of HTD coal samples are 161.6, 149.7, and 164.5 °C, lower than that of RC (177.6 °C). This indicates that the spontaneous combustion tendency of lignite increases after HTD. With the increase in HTD temperature, the *T*_cr_ of coal samples decreases first and then increases. This is consistent with the results from CO_2_ release behavior. The *T*_cr_ of HTD230 is 149.7 °C, indicating that the temperature range 35–140 °C for studying the low-temperature oxidation behaviors of HTD coal samples is reasonable.

Through the above research results, it is found that there are great differences for different coal samples in the release behaviors of CO_2_ and the critical ignition temperature. HTD coal samples have higher low-temperature oxidation activities and spontaneous combustion tendencies than RC. The low-temperature oxidation activity and spontaneous combustion tendency of HTD coal samples first increase and then decrease as the HTD temperature increases.

### 2.2. Effect of HTD on the Low-Temperature Oxidation of the Coal Sample

As listed in Table 1, after HTD, the moisture and volatile matter contents of lignite decrease, and the ash content is fluctuant, resulting from the decomposition of organic matter and the slight dissolution of soluble minerals. As for the elemental composition in organic matter, the carbon increases. HTD affects the composition and structure of lignite. This is why the low-temperature oxidation behaviors of the HTD coal samples change. The factors that affect the low-temperature oxidation of lignite mainly comprise the operating parameters such as temperature and time and the physico-chemical structure parameters such as surface area, pore volume, active groups content. The oxidizing temperature and time are constant, and they are not the main factors in this study. In terms of physico-chemical structure, it has been reported that CO_2_ may be directly generated by the decomposition of intermediates from the oxidation of the aliphatic structure and inherent oxygen-containing functional groups in coal [15,16,17]. In this process, the pores provide a channel for the diffusion of O_2_, and the surface provides sites for the reaction. The aliphatic structure, oxygen-containing functional groups, and pore and surface structure are crucial in the low-temperature oxidation process. Therefore, the physico-chemical structures of coal samples were characterized to explore the effect of HTD on the low-temperature oxidation of lignite.

**Figure 5:**
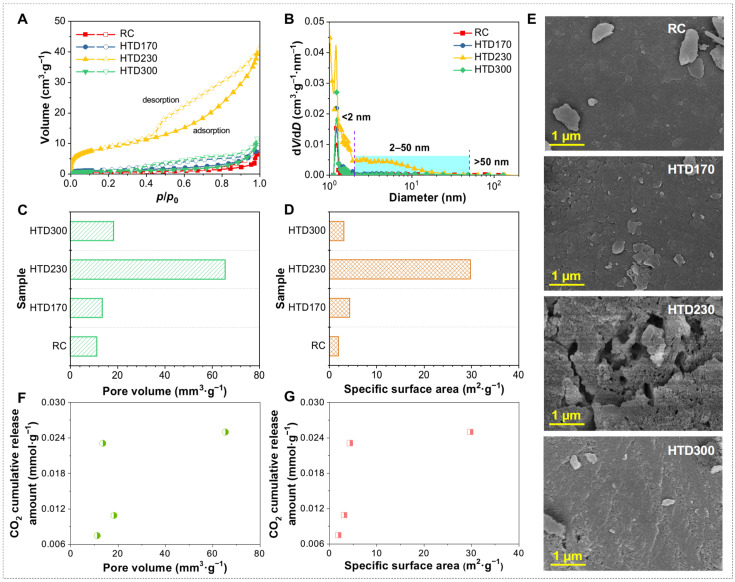
N_2_ adsorption and desorption isotherm (**A**), pore size distribution (**B**), pore volume (**C**), specific surface area (**D**), and SEM pictures (**E**) of RC and HTD coal samples. Relationship between CO_2_ cumulative release amount with pore volume (**F**) and specific surface area (**G**).

The N_2_ adsorption/desorption curves and SEM pictures of RC and HTD coal samples were measured to characterize the change in pore structure and surface morphology. As displayed in Figure 5A, these adsorption and desorption isotherms all have obvious hysteresis loops, and capillary condensation occurs under high relative pressure, indicating that these coal samples all contain mesopores, which can be proved from Figure 5B. It is found from the shape of hysteresis loops that the pore structure in coal is mainly flat or slit shaped (Figure 5A,E). The shape of the hysteresis loops of the coal samples at different temperatures basically does not change, indicating that HTD at different temperatures has little effect on the type of pores. As shown in Figure 5C,D, the pore volume and specific surface area of samples increase first and then decrease after HTD, and the inflection point appears at 230 °C. This is mainly because, at relatively low temperatures (170–230 °C), the pores are developed with the removal of water molecules and the generation of gas from the coal pores, which makes the specific surface area and total pore volume increase. After 230 °C, the specific surface area and pore volume of coal samples decrease significantly as the HTD temperature continues to increase. It is the high temperature that results in the bridge bonds in lignite breaking, thus causing the pore structure to shrink and collapse.

In the process of the low-temperature oxidation of lignite, the O_2_ content diffused to the lignite surface is mainly affected by the pore volume and specific surface area of the coal samples. Therefore, the relationship between the cumulative CO_2_ release amount and the pore volume and specific surface area was explored, as shown in Figure 5F,G. It is found that the cumulative release amount of CO_2_ presents an irregular change with the increase in pore volume and specific surface area. The corresponding pore volume and surface area are linearly fitted to the cumulative CO_2_ release amount, and the correlation coefficients are 0.10 and 0.21, respectively. This manifests as the pore volume and specific surface area having no significant influence on the cumulative CO_2_ release amount during the low-temperature oxidation process at 35–140 °C.

**Figure 6:**
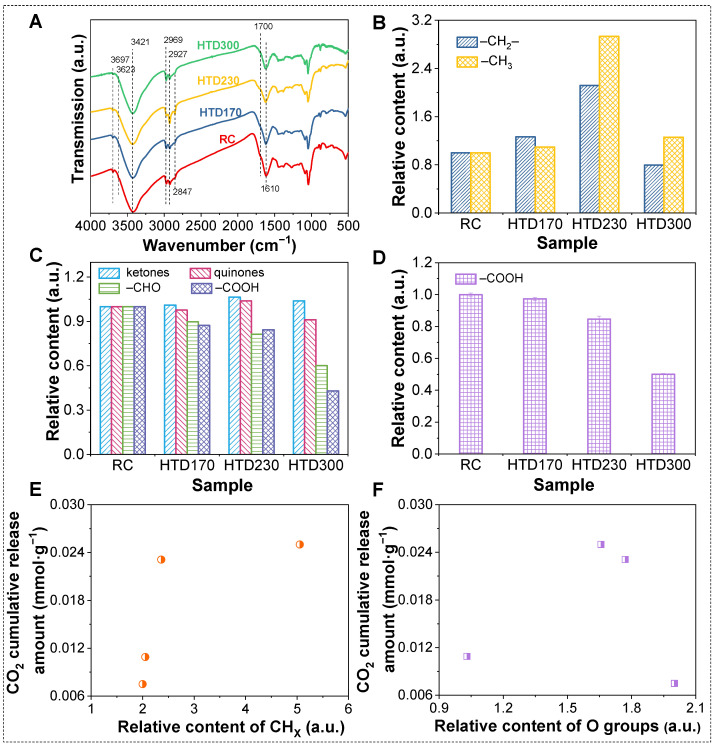
FT-IR spectra of RC and HTD coal samples (**A**). Relative contents of CH_X_ (**B**) and oxygen-containing functional groups (**C**) obtained from FT-IR spectra. Relative content of carboxyl group obtained by chemical titration (**D**). Relationship between CO_2_ cumulative release amount and relative content of CH_X_ (**E**) and oxygen-containing functional groups (**F**).

**Figure 7:**
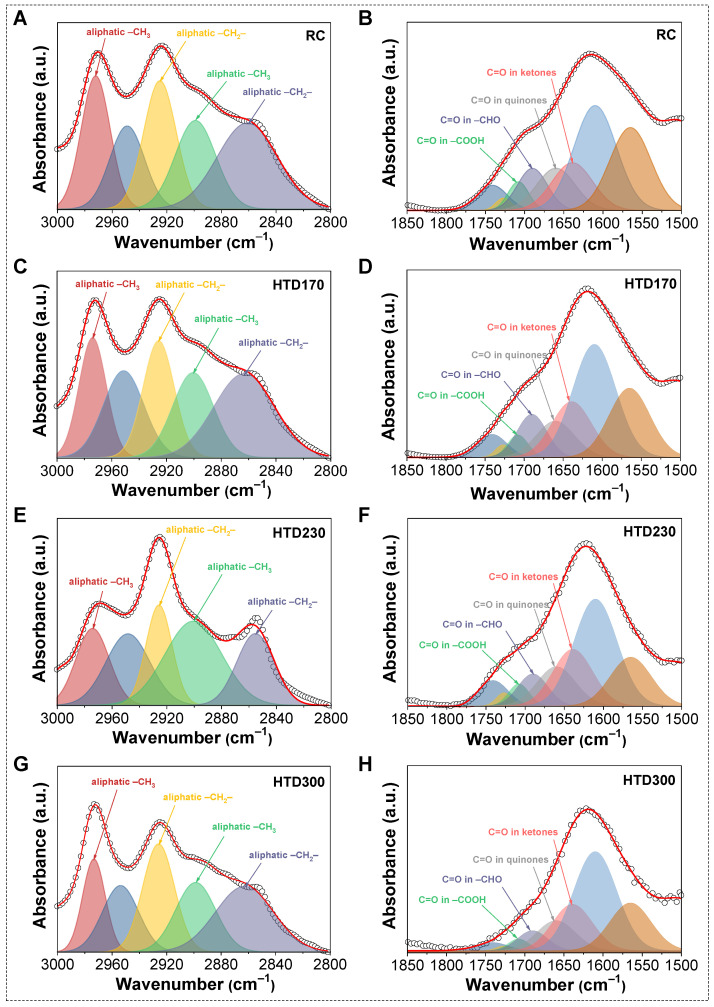
Typical deconvolution for 3000–2800 cm^−1^ zone: (**A**): RC, (**C**): HTD170, (**E**): HTD230, and (**G**): HTD300 and 1850–1500 cm^−1^ zone: (**B**): RC, (**D**): HTD170, (**F**): HTD230, and (**H**): HTD300 of FT-IR.

FT-IR spectra of coal samples are displayed in Figure 6A. It can be seen that the position of the main characteristic peak of the coal samples is basically unchanged after HTD. Then, the quantitative analysis was carried out on the FT-IR spectra of the RC and HTD coal samples, as shown in Figure 7.

The relative contents of functional groups are presented in Figure 6B,C. In order to prove the feasibility of quantitative analysis, the carboxyl group (–COOH) contents of RC and HTD coal samples were determined by chemical titration (Figure 6D). As illustrated in Figure 6C,D, the change trend of the relative content of –COOH in the RC and HTD coal samples obtained from the FT-IR spectra is consistent with that from chemical titration. It is feasible to obtain the relative content change in functional groups by FT-IR spectra analysis.

As can be seen from Figure 6B, the relative contents of –CH_3_ and –CH_2_– ([CH_X_]) increase after HTD and show a trend of first increasing and then decreasing with the increase in HTD temperature. The relative contents of CH_X_ are the highest in HTD230. For the oxygen-containing functional groups in Figure 6C,D, the relative contents of –COOH and –CHO ([O]) in lignite decrease after HTD. With the increase in HTD temperature, the removal rates of –COOH and –CHO show an increasing trend. The changes are not obvious for ketones and quinones. In the process of low-temperature oxidation of lignite, CO_2_ mainly comes from the oxidation of CH_X_ and the decomposition of oxygen-containing functional groups. In order to explore the relationship between the cumulative CO_2_ release amount and functional groups, they were correlated in Figure 6E,F. Both of them are linearly fitted with the cumulative CO_2_ release amount, and their correlation coefficients are as low as 0. With the increase in CH_X_ and oxygen-containing functional groups, the cumulative release amount of CO_2_ presents an irregular change. This shows that there is no obvious relationship between the cumulative CO_2_ release amount and the relative content of CH_X_ and oxygen-containing functional groups.

In summary, for HTD coal samples, there is no good correlation between the cumulative release amount of CO_2_ and the relative contents of functional groups and pore structure parameters during low-temperature oxidation in the temperature range 35–140 °C. This may be because the low-temperature oxidation process of HTD coal samples is carried out in different stages, and the main influencing factors are different at different stages. It is unreasonable to relate the cumulative release amount of CO_2_ with the structure parameters.

### 2.3. Kinetic Analysis of Low-Temperature Oxidation of HTD Coal Samples

According to the above analysis, the low-temperature oxidation process of HTD coal samples is carried out in different stages, and the main influencing factors are different at different stages. In the process of low-temperature oxidation, a change in the main influencing factors would lead to a change in the speed control step, resulting in a change in the activation energy. A change in the chemical reactions would also cause a change in the activation energy. Therefore, the kinetic analysis of the CO_2_ release process during the low-temperature oxidation of lignite was carried out, and the activation energy of CO_2_ release was solved.

**Figure 8:**
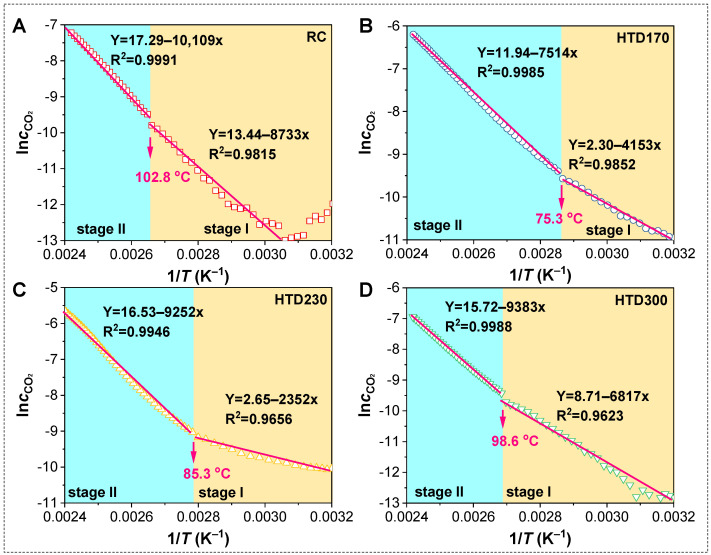
Plots of ln*c*_CO2_ versus 1/*T* in low-temperature oxidation process of RC (**A**), HTD170 (**B**), HTD230 (**C**), and HTD300 (**D**).

Figure 8 shows the relationship between ln*c*_CO2_ and 1/*T* in the low-temperature oxidation process of RC and HTD coal samples. The absolute value of the slope of the line reflects the activation energy. This process is roughly divided into two stages according to the change in activation energy. Therefore, kinetic parameters of different stages for different coal samples are fitted according to Formula (5). The fitting correlation coefficient is as high as 0.96, which indicates that it is appropriate to study the low-temperature oxidation process in two stages. Table 2 summarizes the activation energies at stage I (*E*_α(1)_) and stage II (*E*_α(2)_) and the inflection point temperatures (*T*_in_) of RC and HTD coal samples. For different coal samples, the activation energy of each stage and the inflection point temperature of the two stages are different. In stage I, the activation energies of the coal samples are 72.61, 34.53, 19.55, and 56.68 kJ/mol, respectively. HTD coal samples have lower *E*_α(1)_ values than RC. The *E*_α(1)_ of the HTD coal samples first decrease and then increase with increasing HTD temperature. After stage I, when the coal temperature reaches the inflection point temperature, the coal sample enters stage II. The *T*_in_ of the RC and HTD coal samples are 102.8, 75.3, 85.3, and 98.6 °C, respectively. HTD samples with lower activation energy in stage I can enter stage II at a lower temperature than RC. After reaching stage II, the oxidation accelerates, releasing a large amount of CO_2_. As for the HTD coal samples, they also have lower *E*_α(2)_ values than RC; however, their activation energy shows an increasing tendency with the increase in HTD temperature in stage II.

### 2.4. Correlation and Mechanism Analysis of Low-Temperature Oxidation of HTD Coal Samples

The low-temperature oxidation process (35–140 °C) of HTD coal samples is divided into two stages according to the change in activation energy. The reactions in each stage are different, and their main influencing factors are also different. The main influencing factors of the two stages are explored with correlation analysis. The aliphatic structure, oxygen-containing functional groups, and pore and surface structure of the lignite play important roles in the low-temperature oxidation process. In the process of low-temperature oxidation, O_2_ diffuses to the coal and interacts with the active sites on the coal surface. The pores provide a channel for the diffusion of O_2_ and gas products, and the surface provides sites for the reaction. Therefore, the activation energies at stage I (*E*_α(1)_) and stage II (*E*_α(2)_) are correlated with four parameters, the relative content of the aliphatic structure ([CH_X_]), the relative content of oxygen-containing functional groups ([O]), the pore volume (*V*), and the specific surface area (*S*), with Spearman correlation analysis.

**Figure 9:**
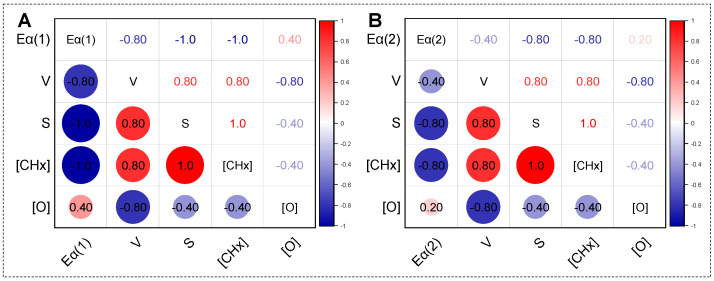
Correlation between activation energy with structure parameters for stage I (**A**) and stage II (**B**).

As depicted in Figure 9A, the Spearman correlation coefficients between *E*_α(1)_ and *V*, *S*, [CH_X_] and [O] are −0.80, −1.0, −1.0, and 0.40, respectively. *E*_α(1)_ is most correlated with the relative content of the aliphatic structure and the specific surface area. In stage I, since the reaction temperature is low, the coal sample generates a small amount of gas (Figure 2) and releases a small amount of heat (Figure 3). The differences in the sample structures lead to the variations in their activation energies during the low-temperature oxidation process. The functional groups on the coal surface act as adsorption sites, such as –CH_3_, –CH_2_–, –CHO, –COOH, and other functional groups [33,34]. Oxygen-containing functional groups cannot decompose at low temperatures. So, in stage I of low-temperature oxidation, the active groups are –CH_3_ and –CH_2_–, which might adsorb O_2_ to produce hydroperoxides and peroxides. As for the pore structure, the adsorption of O_2_ in this stage occurs on the surface of the samples, and a larger pore volume promotes faster diffusion of O_2_ to the coal surface. Therefore, the main factors in this stage are the aliphatic structure and surface structure. The activation energy in stage I decreases with the increase in [CH_X_] and *S*. In other words, HTD samples with more CH_X_ and a large surface area are more prone to oxidation. Since the low-temperature oxidation reaction in this stage is mainly a process of adsorption of oxygen by aliphatic groups, stage I can be described as the oxygen adsorption stage.

For stage II, as seen in Figure 9B, the correlations between *E*_α(2)_ and *V*, *S*, [CH_X_], and [O] (−0.40, −0.80, −0.80, and 0.20, respectively) are relatively weak. In this stage, since the reaction temperature rises, a large amount of gas begins to be released, and the oxidation reaction becomes intense (Figure 2). The coal sample releases more heat, reducing the temperature difference between the center temperature and the surrounding temperature (Figure 3). The production of CO_2_ might be generated by the thermal decomposition of surface oxides and the inherent oxygen-containing functional groups. The surface oxides come from the adsorption of O_2_ via the aliphatic structure on the sample surface in stage I. The *V*, *S*, and [CH_X_] of the HTD coal samples first increase and then decrease, while [O] decreases with increasing HTD temperature (Figure 5 and Figure 6). The increase in *V*, *S*, and [CH_X_] for HTD170 and HTD230 would promote the oxidation reaction; however, a decrease in [O] is not conducive. Changes in *V*, *S*, [CH_X_], and [O] have different effects on low-temperature oxidation. This change in activation energy in stage II is caused by competition between four parameters. Based on this, the HTD coal samples exhibit complex CO_2_ release behavior in stage II. Since a series of reactions began to produce more gaseous products, stage II can be defined as the accelerated oxidation stage.

**Figure 10:**
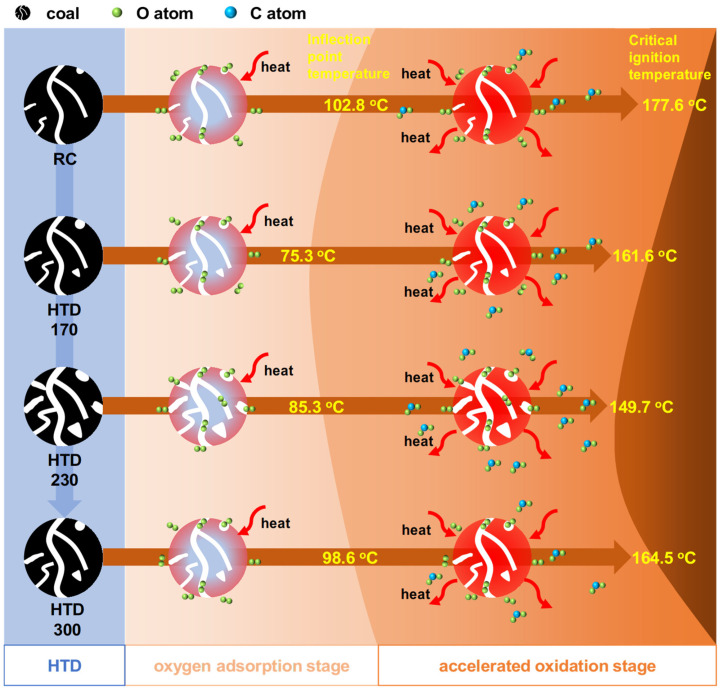
Low-temperature oxidation process of HTD coal samples.

Based on the above discussion, the low-temperature oxidation process of the HTD coal samples is summarized in Figure 10. To sum up, the low-temperature oxidation process (35–140 °C) is divided into two stages, the oxygen adsorption stage and the accelerated oxidation stage. The oxygen adsorption stage is controlled by the aliphatic and surface structures, and the accelerated oxidation stage is affected by the competition of physico-chemical structures. The low-temperature oxidation in stage I promotes the reaction in stage II. In order to reduce the low-temperature oxidation activity of HTD coal samples, measures that reduce the CH_X_ content of the coal sample and decrease the surface area should be taken to minimize the probability of oxygen adsorption reactions.

## 3. Materials and Methods

### 3.1. Preparation of HTD Coal Sample

The lignite sample was collected from Inner Mongolia in China. It was ground and sieved to a particle size of 0.425–0.850 mm under an N_2_ atmosphere to minimize the possible moisture loss and structure damage and was then stored in a brown reagent bottle for subsequent use. The total moisture content in the raw coal (RC) was determined as 32.32% according to the Chinese standard GB/T 211-2017 [35].

The hydrothermal dewatering (HTD) experiments were carried out in a 1 L autoclave. The mixture of 147 g lignite (100 g in dry basis) and 153 g deionized water at a 1:2 proportion of dry coal to water were added to the autoclave and heated to a desired temperature (170, 230, or 300 °C) for 30 min. Finally, HTD coal samples were obtained by filtering and drying after cooling to room temperature. The coal samples hydrothermally dewatered at different operating temperatures were marked with HTD170, HTD230, and HTD300, respectively (Figure 1A). The schematic diagram of the autoclave and the specific operation procedure can be obtained in our previous study [32]. The proximate and ultimate analyses of RC and HTD coal samples are provided in Table 1. The experiment was repeated at least 3 times to ensure good reproducibility.

### 3.2. Low-Temperature Oxidation of Coal Sample

A device was designed to investigate the gas release behavior in the low-temperature oxidation process of lignite, as shown in Figure 1B. Firstly, 15.0 g of coal sample was loaded into the reactor, and a gaseous mixture of Ar and O_2_ with volume proportions of 79% and 21% (air simulated by Ar and O_2_) was passed. The exhaust gas was detected by an online mass spectrometer (MS). After the MS signal was stabilized, the coal sample was heated from room temperature to 140 °C at a heating rate of 2 °C/min. The released amounts of CO and CO_2_ were determined in this process. It should be noted that all coal samples were pre-treated with a vacuum to remove impurities and moisture before the experiments.

A wire mesh reactor made of stainless-steel mesh (25 × 25 × 25 mm^3^) was used to monitor the temperature change in the coal sample in the low-temperature oxidation process and to test the spontaneous combustion tendency. As shown in Figure 1C, 10.0 g of coal sample was loaded into the wire mesh reactor. Three thermocouples were inserted into the reactor. One thermocouple was placed at the geometric center of the coal sample, and the other two thermocouples were placed at the left and right sides 6.3 mm away from the center. The coal sample was heated from room temperature to a desired temperature at a heating rate of 20 °C/min, and the temperatures of the coal samples (*T*_1_, *T*_2_, and *T*_3_) and the oven (*T*_o_) were recorded in real time. The critical ignition temperature (*T*_cr_) of the coal sample was determined by changing the termination temperature of the oven.

### 3.3. Analysis Methods

#### 3.3.1. Characterization of Coal Sample

The pore volume and specific surface area of the coal sample were measured by an N_2_ adsorption analyzer (JW-BK122W, Beijing JWGB Sci. &Tech. Co., Ltd., Beijing, China). N_2_ adsorption and desorption isotherms of the coal sample were measured at 77 K with the relative pressure (*p*/*p*_0_) ranging from 0.01 to 0.99. The pore volume was obtained by the Barret–Joyner–Halenda (BJH) model. The specific surface area was obtained by the Brunauer–Emmet–Teller (BET) formula.

An emission scanning electron microscope (SEM, Czech TESCAN, MAIA3 LMH, Brno, Czech Republic) was used to scan the surface morphology characteristics of the coal sample. The magnification ratio was 20,000, and the voltage was 5.0 kV.

The functional groups of the coal sample were characterized by a Fourier transform infrared (FT-IR) spectrometer (INVENIO R, BRUKER, Munich, Germany). A 1.0 mg coal sample and 100.0 mg KBr were accurately weighed and fully ground in an agate mortar. The mixture was pressed to make a 13 mm wafer. The spectrum of the coal sample was measured in a wavenumber range of 4000–400 cm^−1^ with a resolution of 4 cm^−1^ from 64 scans. The assignments in the 3000–2800 cm^−1^ and 1850–1500 cm^−1^ zones were listed in Table 3.

The content of the carboxyl group (–COOH) in the coal sample was determined by the chemical titration method. About 1.0 g of the coal sample was accurately weighed and immersed in a conical bottle containing 20 mL of NaHCO_3_ solution with a concentration of 0.11 mol/L for 48 h to make it fully react. After the reaction, the mixture of water and the coal sample was filtered, and the clarified filtrate was obtained. The excessive content of NaHCO_3_ in the filtrate was titrated with HCl. The difference between the amount of NaHCO_3_ substance added and the amount of HCl substance consumed was the content of –COOH on the sample surface.

#### 3.3.2. Kinetic Analysis Method

In the low-temperature oxidation process, coal reacts with oxygen molecules as follows.

Coal + O_2_ → *m* CO + *g* CO_2_ + other products

According to chemical reaction theory and Arrhenius equation, the coal oxygen reaction rate can be expressed by the following formula [39,40]:

(2) $$v\left(T\right)=v\left(O_{2}\right)=\frac{v\left(CO\right)}{m}=\frac{v\left({CO}_{2}\right)}{g}=Ac_{O_{2}}^{n}\exp(-\frac{E_{\alpha }}{RT})$$

where *v* is the reaction rate; *T* is the temperature of coal (K); *A* is the pre-exponential factor; *c*_O2_ is the amount of O_2_ in the reaction gas; *n* is the reaction order; *E*_α_ is the activation energy (J·mol^−1^); *R* is the molar gas constant (8.314 J·mol^−1^·K^−1^).

Assuming that the gas flows only along the axial direction of the reactor, the CO_2_ generation rate of the coal sample along the axial d*x* of the reactor can be calculated with Formula (3).

(3) $$Sv\left({CO}_{2}\right)dx=kv_{g}dc$$

where *S* is the cross-sectional area of the reactor (m^2^); *k* is the unit conversion factor; *v*_g_ is the airflow rate (m^3^·s^−1^); *c* is CO_2_ production.

From Formulas (2) and (3), Formula (4) can be obtained.

(4) $$ASgc_{O_{2}}^{n}exp\;(-\frac{E_{\alpha }}{RT})dx=kv_{g}dc$$

Formula (5) can be obtained by integrating Formula (4).

(5) $$lnc_{{CO}_{2}}=\ln\left(\frac{ASLgc_{O_{2}}^{n}}{kv_{g}}\right)-\frac{E_{\alpha }}{RT}$$

where *L* is the length of reactor (m); *c*_CO2_ is the CO_2_ concentration.

The plots for ln*c*_CO2_ versus 1/*T* yield a straight line. The apparent activation energy (*E*_α_) was obtained from the slope of the straight line.

#### 3.3.3. Correlation Analysis Method

Spearman correlation coefficient is a nonparametric index that measures the dependence of two variables. It uses monotone formulas to evaluate the correlation of two statistical variables. The Spearman correlation coefficient was calculated by using the following formula:

(6) $$\rho =\frac{\sum_{i=1}^{n}\left(x_{i}-\bar{x})(y_{i}-\bar{y}\right)}{\sqrt{\sum_{i=1}^{n}{\left(x_{i}-\bar{x}\right)}^{2}}\sqrt{\sum_{i=1}^{n}{\left(y_{i}-\bar{y}\right)}^{2}}}$$

where *ρ* is the Spearman correlation coefficient whose value ranges from −1 ≤ *ρ* ≤ 1. When *ρ* < 0, it is a negative correlation; when *ρ* > 0, it is a positive correlation; there is no correlation when *ρ* = 0. The greater the absolute value of the correlation coefficient, the closer the correlation between the two variables.

## 4. Conclusions

Through CO_2_ release, temperature change, kinetic analysis, and correlation analysis, the effect mechanism of HTD on low-temperature oxidation in the temperature range of 35–140 °C were investigated. The following conclusions are drawn.

(1) HTD has a great effect on the low-temperature oxidation of lignite. Based on the change in the CO_2_ cumulative release amount and critical ignition temperature, HTD coal samples have higher low-temperature oxidation activities and spontaneous combustion tendencies than RC, and the low-temperature oxidation activity and spontaneous combustion tendency of HTD coal samples first increase and then decrease with increasing HTD temperature;

(2) According to the change in activation energy, the low-temperature oxidation process (35–140 °C) is divided into two stages, the oxygen adsorption stage and the accelerated oxidation stage. Based on the results of the correlation analysis, the main factor affecting low-temperature oxidation of the HTD coal samples is determined as the aliphatic structures and the specific surface area for the oxygen adsorption stage; meanwhile, the competition between physico-chemical structures is the main factor for the accelerated oxidation stage. In order to reduce the low-temperature oxidation activity and spontaneous combustion tendency, the CH_X_ content and specific surface area should be decreased to prevent oxygen adsorption. Therefore, further research should be focused on methods for decreasing the spontaneous combustion behavior of lignite by the regulation of the coal structure.

## Figures and Tables

**Table 1:** Proximate and ultimate analyses of RC and HTD coal samples.

Sample	Proximate Analysis (%)			Ultimate Analysis (%, daf)				
	M_ad_	A_d_	V_daf_	C	H	O *	N	S
RC	12.35	12.73	43.88	73.60	4.21	19.16	1.13	1.90
HTD170	11.76	14.16	43.98	74.12	3.58	20.05	1.15	1.10
HTD230	10.11	14.16	42.42	74.74	3.43	19.69	1.10	1.04
HTD300	7.43	12.16	38.31	76.69	3.17	17.82	1.23	1.09

Note: M, moisture; A, ash; V, volatile matter; ad, air dried basis; d, dry basis; daf, dry ash-free basis; * by difference.

**Table 2:** Fitting parameters of RC and HTD coal samples in two stages of low-temperature oxidation.

Sample	*E*_α(1)_ (kJ/mol)	*E*_α(2)_ (kJ/mol)	*T*_in_ (℃)
RC	72.61	84.05	102.8
HTD170	34.53	62.47	75.3
HTD230	19.55	76.92	85.3
HTD300	56.68	78.01	98.6

**Table 3:** Assignment of FT-IR bands in wavenumber range of 3000–2800 cm^−1^ and 1850–1500 cm^−1^.

Wavenumber (cm^−1^)	Assignment	Reference
2962	Aliphatic –CH_3_ asymmetric stretching vibration	[33,36,37]
2925	Aliphatic –CH_2_– asymmetric stretching vibration	[33,36,37]
2895	Aliphatic C–H stretching vibration	[33,36,37]
2872	Aliphatic –CH_3_ symmetric stretching vibration	[33,36,37]
2850	Aliphatic –CH_2_– symmetric stretching vibration	[33,36]
1815	Open chain fatty anhydride vibration	[38]
1785	Cyclic aliphatic anhydride vibration	[38]
1745	C=O vibration in aliphatic esters	[27]
1725	C=O vibration in aromatic esters	[27]
1710	C=O vibration in carboxyl groups –COOH	[33]
1690	C=O vibration in aldehyde groups –CHO	[38]
1660	C=O vibration in quinones	[38]
1610	Aromatic hydrocarbons C=C vibration	[33,36,37]

## Data Availability

The original contributions presented in this study are included in this article; further inquiries can be directed to the corresponding authors.
